# Supplementary material for: Polydextrose with and without Bifidobacterium animalis ssp. lactis 420 drives the prevalence of Akkermansia and improves liver health in a multi-compartmental obesogenic mice study
Source: PLoS One. 2021 Dec 2;16(12):e0260765. doi: 10.1371/journal.pone.0260765 (PMC8638982; doi:10.1371/journal.pone.0260765)
Supplement: S1 Fig — (A) Fecal samples at week 0. (B) Fecal samples at week 3. The order of the treatments is from left to right: NC, HFD, HFD+B420, HFD+PDX and HFD+S. (PDF) [file pone.0260765.s001.pdf]

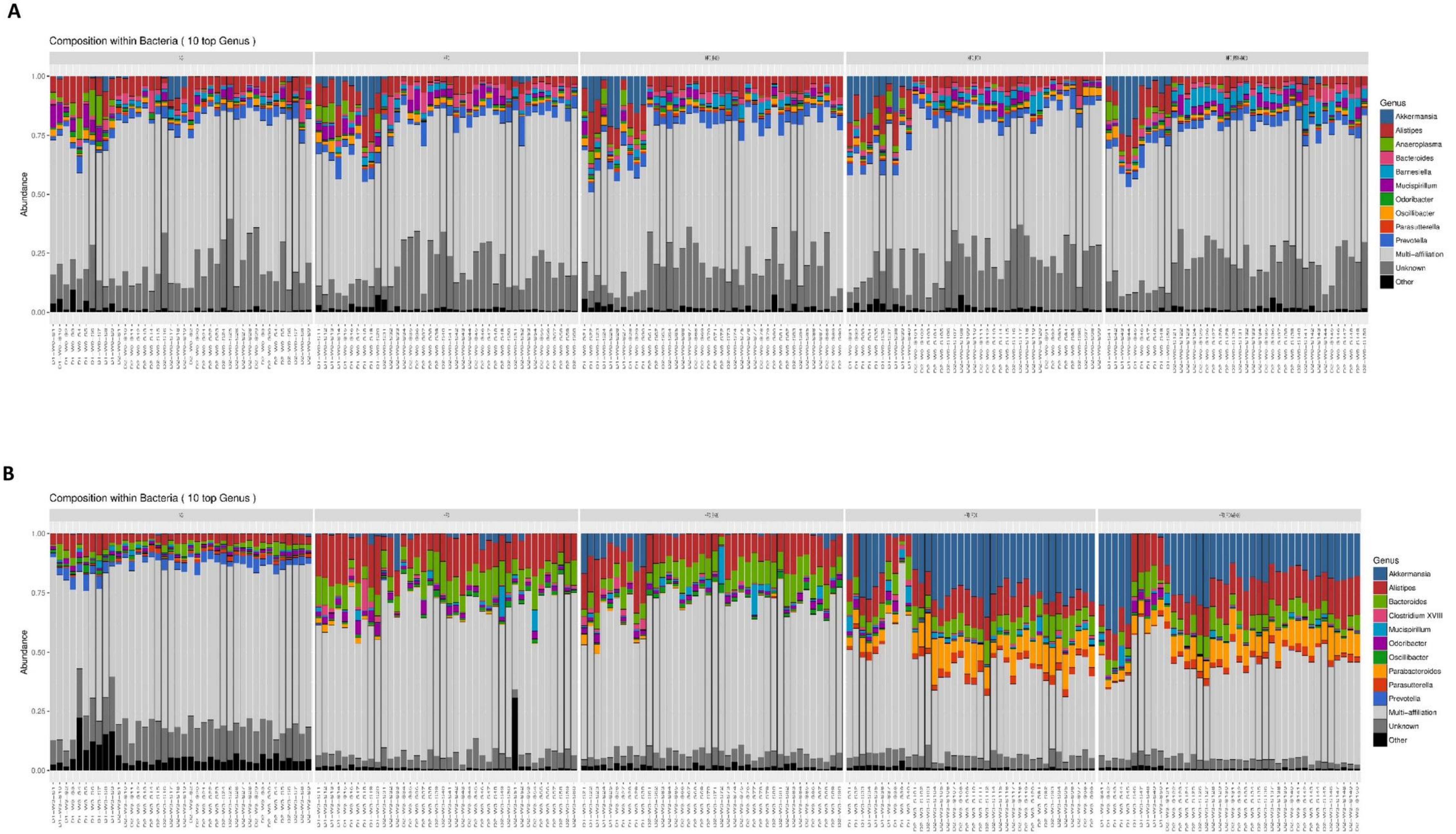

S1 Fig. Relative abundance at Genus level from metagenomic sequencing. (A) Fecal samples at week 0. (B) Fecal samples at week 3. The order of the treatments is from left to right: NC, HFD, HFD+B420, HFD+PDX and HFD+S.

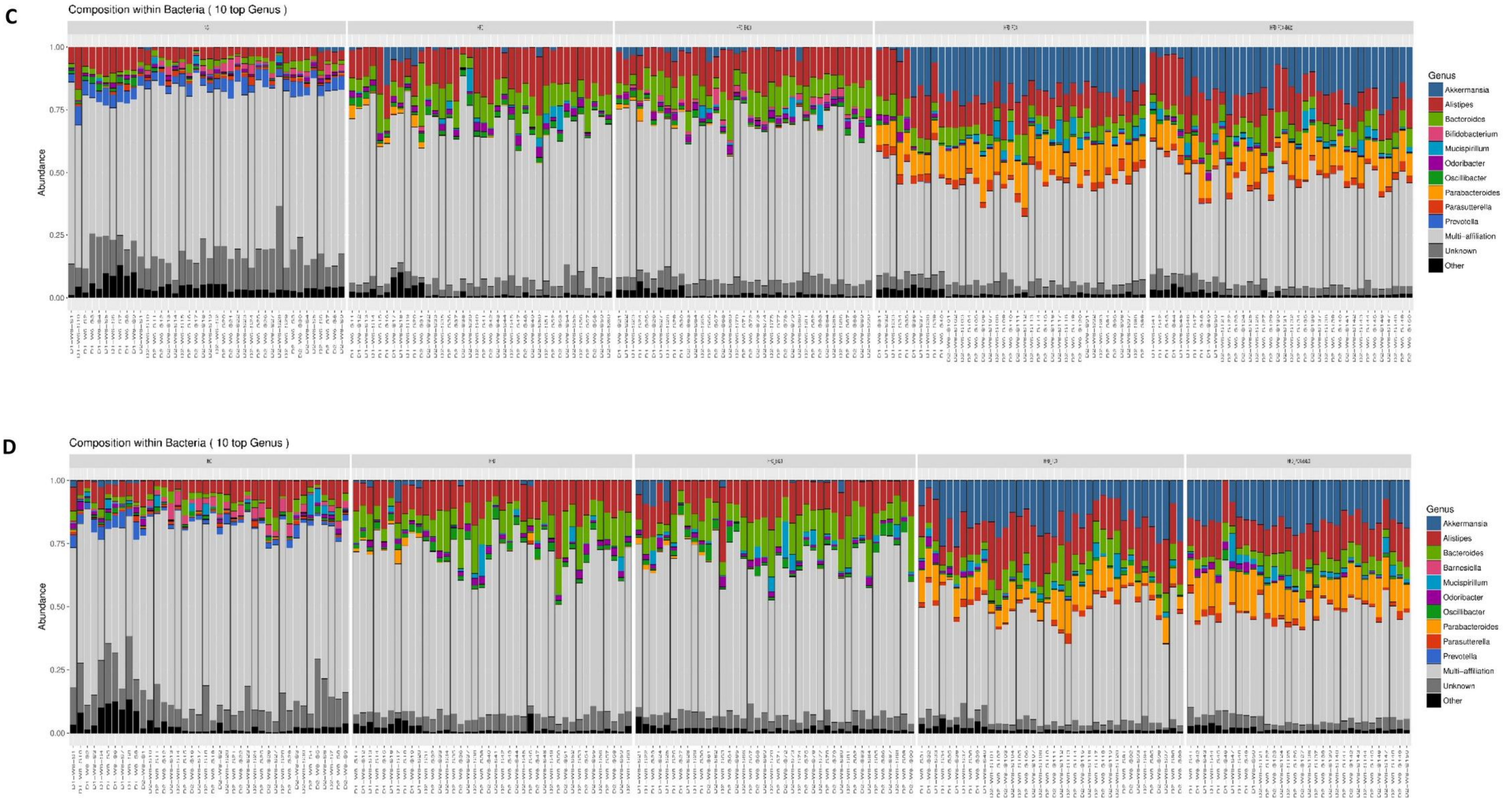

S1 Fig. Relative abundance at Genus level from metagenomic sequencing. (C) Fecal samples at week 6. (D) Fecal samples at week 8. The order of the treatments is from left to right: NC, HFD, HFD+B420, HFD+PDX and HFD+S.

E

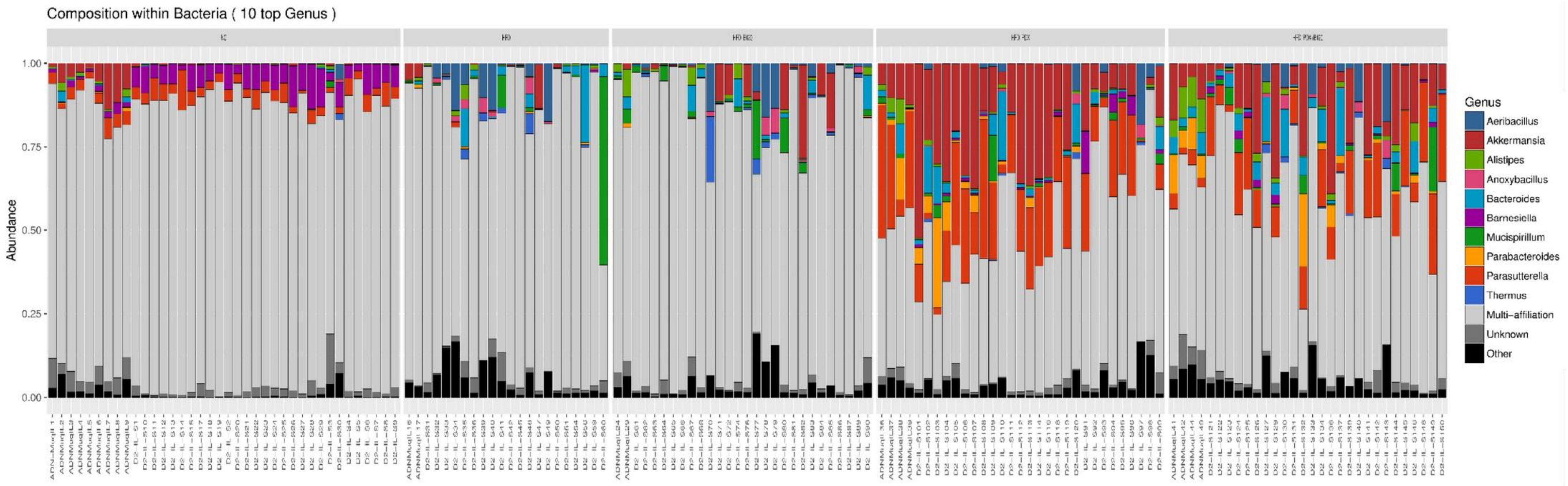

F

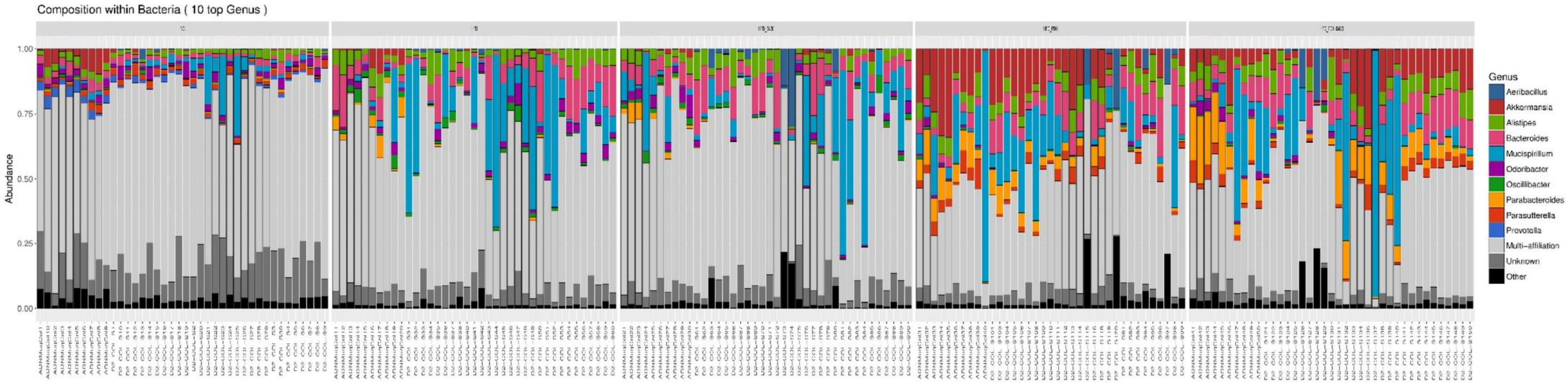

S1 Fig. Relative abundance at Genus level from metagenomic sequencing. (E) Ileum samples. (F) Colon samples. The order of the treatments is from left to right: NC, HFD, HFD+B420, HFD+PDX and HFD+S.

G

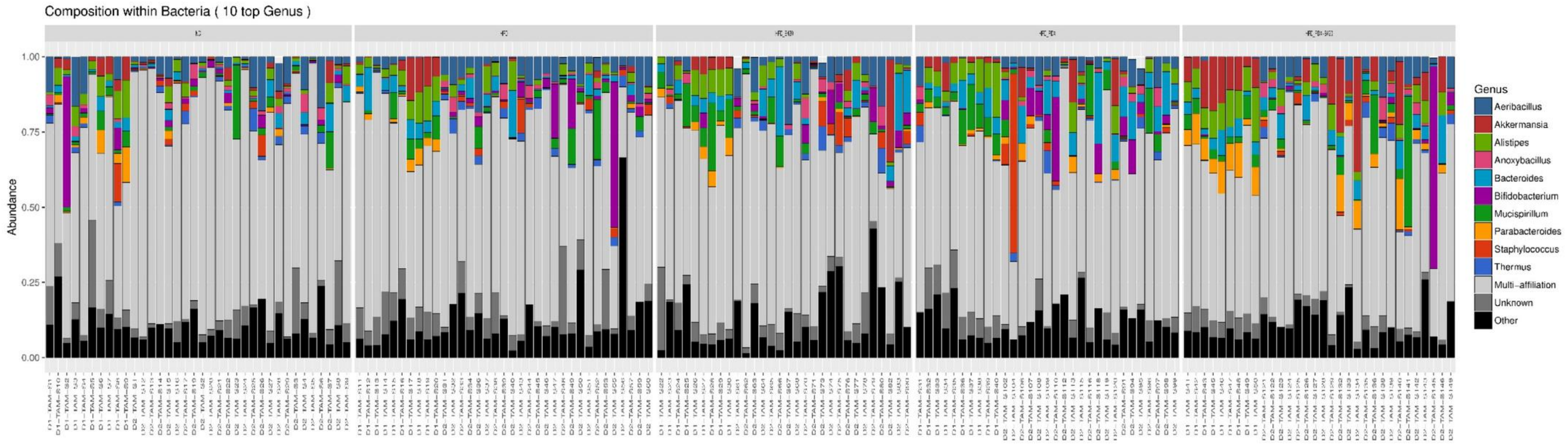

S1 Fig. Relative abundance at Genus level from metagenomic sequencing. (G) Adipose tissue (MAT) samples. The order of the treatments is from left to right: NC, HFD, HFD+B420, HFD+PDX and HFD+S.
