# Supplementary material for: Polydextrose with and without Bifidobacterium animalis ssp. lactis 420 drives the prevalence of Akkermansia and improves liver health in a multi-compartmental obesogenic mice study
Source: PLoS One. 2021 Dec 2;16(12):e0260765. doi: 10.1371/journal.pone.0260765 (PMC8638982; doi:10.1371/journal.pone.0260765)
Supplement: S2 Fig — (A) Fecal samples at week 0. (B) Fecal samples at week 8. (C) Ileum samples. (D) Colon samples. (E) Adipose tissue (MAT). (PDF) [file pone.0260765.s002.pdf]

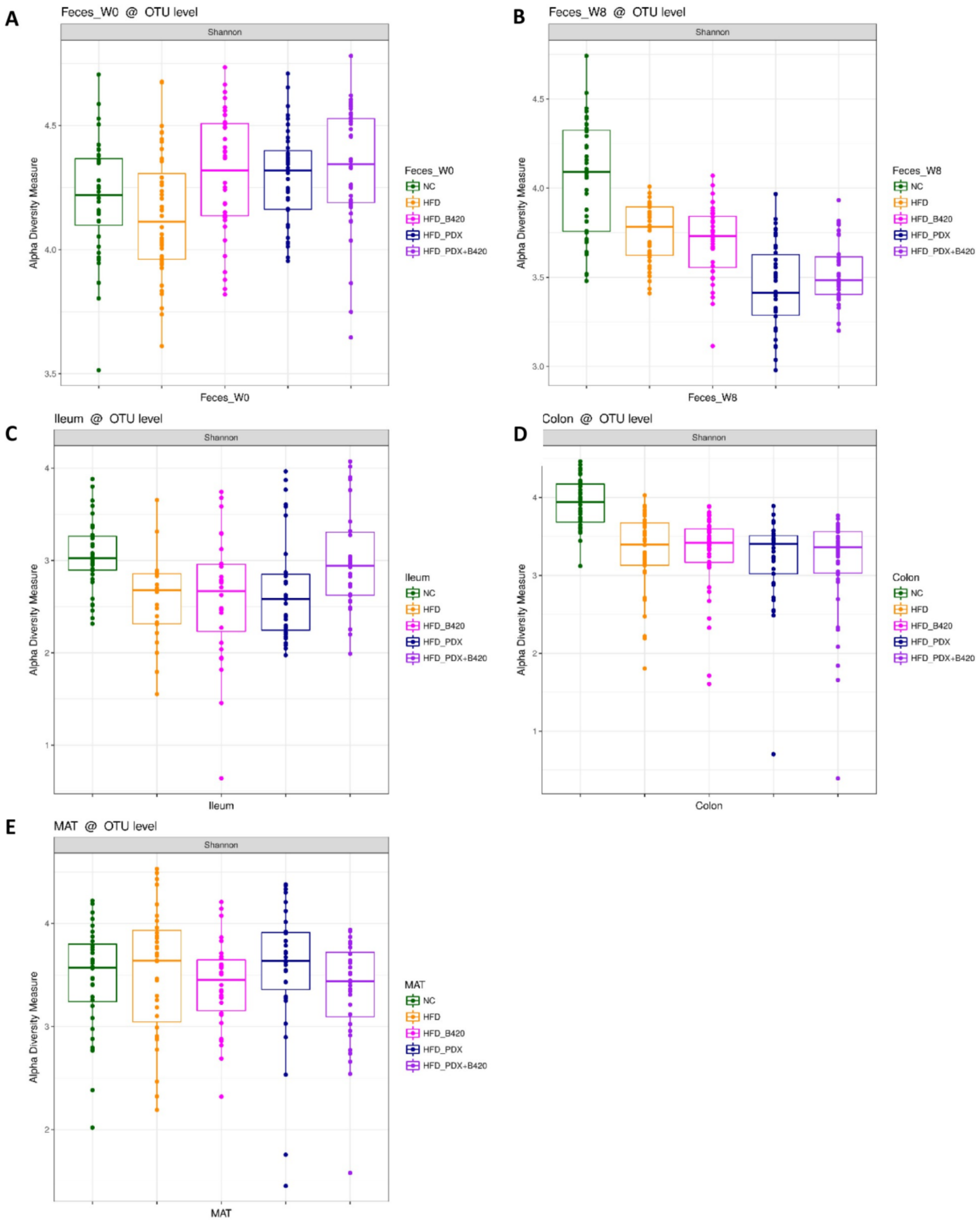

S2 Fig. Microbiota α-diversity index evaluated using the Shannon index. (A) Fecal samples at week 0. (B) Fecal samples at week 8. (C) Ileum samples. (D) Colon samples. (E) Adipose tissue (MAT).
