# Supplementary material for: Polydextrose with and without Bifidobacterium animalis ssp. lactis 420 drives the prevalence of Akkermansia and improves liver health in a multi-compartmental obesogenic mice study
Source: PLoS One. 2021 Dec 2;16(12):e0260765. doi: 10.1371/journal.pone.0260765 (PMC8638982; doi:10.1371/journal.pone.0260765)
Supplement: S3 Fig — (A) Fecal samples at week 0. (B) Fecal samples at week 8. (C) Ileum samples. (D) Colon samples. (E) Adipose tissue (MAT) samples. (PDF) [file pone.0260765.s003.pdf]

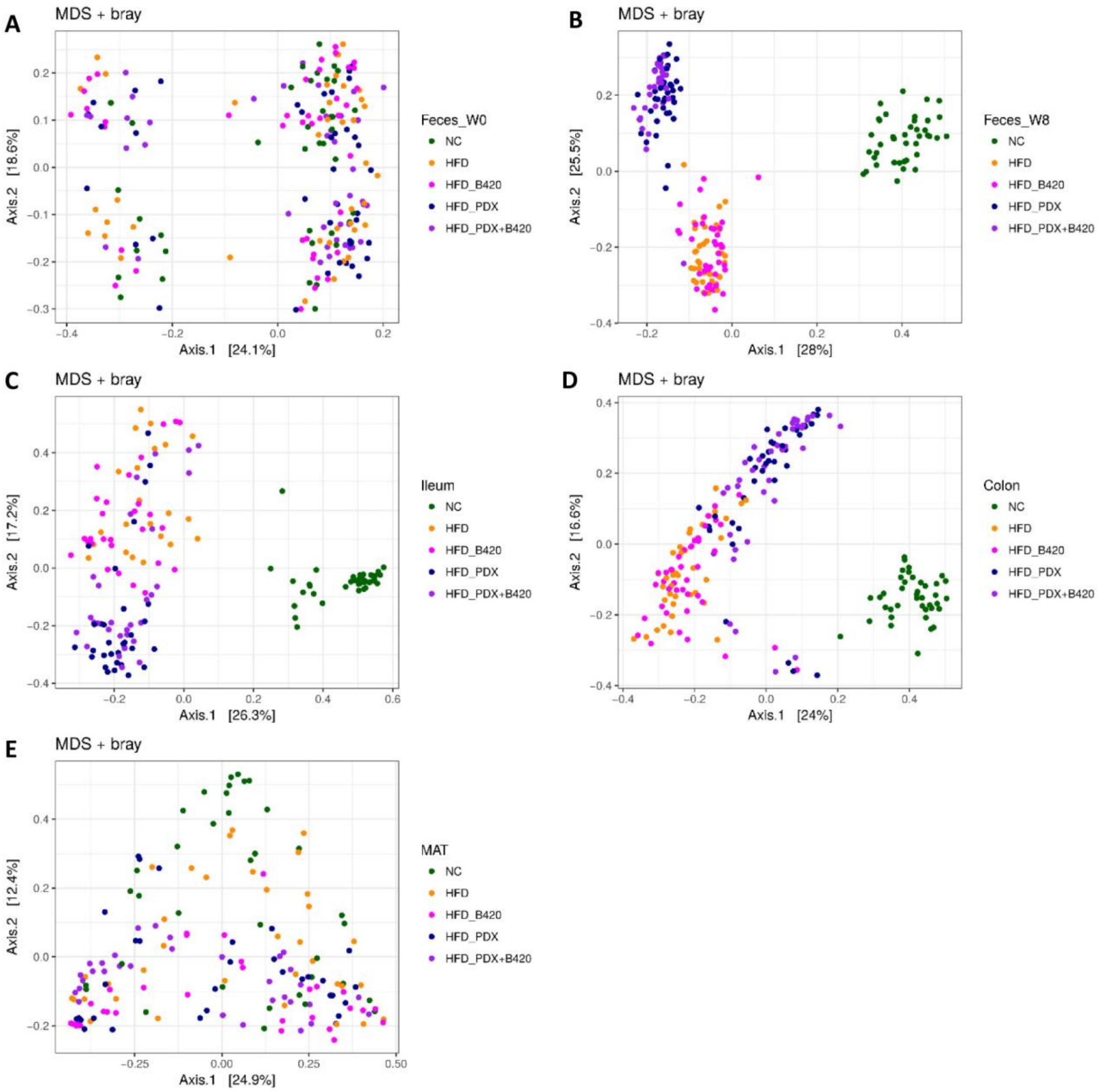

S3 Fig. Principal coordinate analysis (PCoA) on microbiota β-diversity clustering based on Bray-Curtis, Jaccard, Unifrac and Weighted Unifrac distances for comparison of the different treatments. (A) Fecal samples at week 0. (B) Fecal samples at week 8. (C) Ileum samples. (D) Colon samples. (E) Adipose tissue (MAT) samples.
