# Supplementary material for: Polydextrose with and without Bifidobacterium animalis ssp. lactis 420 drives the prevalence of Akkermansia and improves liver health in a multi-compartmental obesogenic mice study
Source: PLoS One. 2021 Dec 2;16(12):e0260765. doi: 10.1371/journal.pone.0260765 (PMC8638982; doi:10.1371/journal.pone.0260765)
Supplement: S4 Fig — (A) Fecal samples. (B) Intact liver samples. (PDF) [file pone.0260765.s004.pdf]

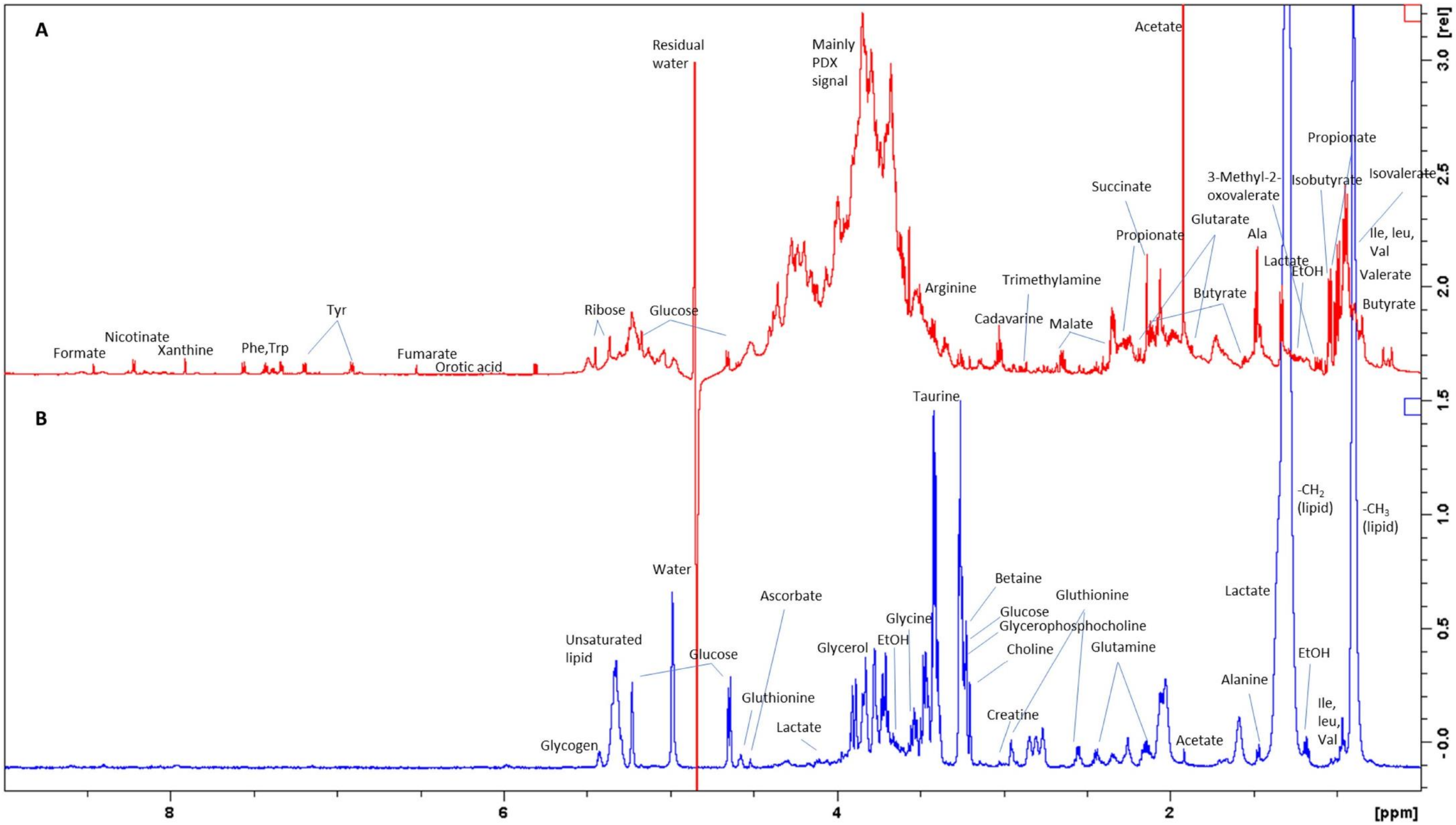

S4 Fig. Representative 1H NMR spectra from the same synbiotic HFD+PDX+B420 mice. (A) Fecal samples. (B) Intact liver samples.
