# Supplementary material for: Polydextrose with and without Bifidobacterium animalis ssp. lactis 420 drives the prevalence of Akkermansia and improves liver health in a multi-compartmental obesogenic mice study
Source: PLoS One. 2021 Dec 2;16(12):e0260765. doi: 10.1371/journal.pone.0260765 (PMC8638982; doi:10.1371/journal.pone.0260765)
Supplement: Attachment — Submitted filename: Response to Reviewers_PONE-D-21-07582R1.docx [file pone.0260765.s005.docx]

# Rebuttal letter (PONE-D-21-07582R1)

Thank you again for the second revision of our manuscript and providing valuable input for improving the manuscript. Below, we have addressed the remaining comments and suggestions of the reviewers.

Journal Requirements:

Please review your reference list to ensure that it is complete and correct. If you have cited papers that have been retracted, please include the rationale for doing so in the manuscript text, or remove these references and replace them with relevant current references. Any changes to the reference list should be mentioned in the rebuttal letter that accompanies your revised manuscript. If you need to cite a retracted article, indicate the article’s retracted status in the References list and also include a citation and full reference for the retraction notice.

No cited papers have been retracted.

Reviewer's Responses to Questions

Comments to the Author

6. Review Comments to the Author

Please use the space provided to explain your answers to the questions above. You may also include additional comments for the author, including concerns about dual publication, research ethics, or publication ethics. (Please upload your review as an attachment if it exceeds 20,000 characters)

Reviewer #1: Thank you very much for the revised manuscript. All of my comments have been addressed satisfactorily.

Nonetheless, there are some minor formatting issues to be corrected. For example, the 'author' of the first reference should be 'OECD', not '2019 O'.

Thank you, this has been amended accordingly.

Reviewer #2: Clement Yde et al. present an impressive assessment of murine metabolomics and metagenomics following five (4+control) different treatment combinations of prebiotic and probiotic elements alongside a high-fat diet. The study is well-written and conveys an interesting message, whereas its methods section comprehensively describes all implemented modalities. The authors have provided extensive evaluations of murine microbiota both at treatment initiation and study endpoint, showcasing relative similarities in diversity indices upon initiation, which is important given the numerous relevant confounding factors (cage, bedding, basal animal conditions etc.) The metabolomic arm could be ideally supplemented with the inclusion of biopsy images from tissues obtained and the analysis and discussion of liver histology and/or immunohistochemistry pertaining to non-alcoholic fatty liver disease.

I fully agree, it would be of great relevance to incl. liver histology in the study. It has been previously been discussed in the response to the first review of the manuscript. In the discussion, it has been stated as an important focus area for future research: “However, further studies are needed to clarify the effect on liver health incl. e.g. histology and hepatic in-vitro models”.

In its current form the manuscript is well delineated and the reviewer believes the authors should follow the suggestions below. However, if available, the inclusion of data on murine body composition and plasma concentrations of insulin, liver enzymes, triglycerides, conventionally-measured cholesterol fractions etc would be of great scientific interest, since it could ascertain the precise pathobiological fingerprint of all described interventions that reflect the altered metabolomic profiles and their correlation with microbiota.

In the clinical trial described in the Stenman et al. paper (EBioMedicine 13 (2016) 190–200; ref. [11]), we report serum insulin, ASAT, ALAT, gamma-glutamyltransferase, total cholesterol, LDL, HDL and triglycerides etc.. Unfortunately, these standard clinical analyses were not incl. in the present study as the main objective was to examine the underlying mechanisms of PDX and B420 treatments explaining the earlier findings in animal studies and human clinical trials on metabolic health.

Additionally, some further suggestions are in order to improve overall readability and quality.

• Consider describing the full dietary energy composition of the murine HFD (total calories, %carbs, %protein, %fat)

The caloric information of protein, carbohydrate and energy density have been added to the ‘Animals and study design’ section.

• Was there any attrition / mice deaths before study completion?

One mouse died at intervention week 7 prior to completion of the study. This has been added to the ‘Animals and study design’ section.

• Gut microbiome constitutes a primary driver of hepatic steatosis and inflammation, both components of non-alcoholic fatty liver disease, whereas relevant interventions through probiotic administration are being discussed across the literature as potential therapeutic strategies (Meroni et al., Nutrients . 2019 Nov 4;11(11):2642; Delzenne et al., Nat Med 2018; 24: 906–907; Le Roy et al., Gut 2013; 62(12): 1787-94) Consider expanding your discussion and thus further abridging the two main areas of your study (microbiota + metabolomics) towards this direction, and to address the functional significance and potential long-term clinical relevance of your findings regarding liver metabolomics, especially considering weight gain differences between human and murine models as described in your already published work, already cited (Ref. 11), and the similarities or differences of metabolomic findings observed herein to recently published evidence on murine NAFLD therapeutics regarding liraglutide (especially relevant owing to the known PDX-induced increase of GLP-1) /elafibranor/empagliflozin administration in HFD-fed mice with HFD-induced non-acoholic steatohepatitis (Perakakis et al., Liver Intl 2021 Aug;41(8):1853-1866).

Thanks for providing very relevant and interesting literature regarding probiotics potential as a therapeutic strategy towards NAFDL. Indeed, I agree that it is relevant to discuss the similarities with effects of liraglutide and elafibranor.

Added to the discussion: “A recent paper by Perakakis et. al [63] describes the underlying mechanistic effect of liraglutide and elafibranor, which are a GLP-1 receptor analog and a dual PPAR α/δ agonist, respectively, in regard to treatment of non-alcoholic fatty liver disease (NAFLD). Striking similarities between the two studies were found for HFD+PDX and HFD+S groups with liraglutide by restoring the levels of glycogen and increasing betaine concentration in the liver. Additionally, both HFD+PDX and HFD+S showed similar results with elafibranor treatment considering the increased glutathione levels in the liver. It has previously been discussed in literature that probiotics supplementation is a potential therapeutic strategy towards NAFDL [64]. Thus, administration of PDX alone or in combination with B420 could potentially be a supplement to liraglutide and elefibranor to be used in the treatment of NAFLD.”

• Consider providing an additional image, embedded in Figure 5, in the form of a time scale graph/diagram showcasing the study design, randomization and number of total mice per group at study end, and measurements per week

To enhance the overview, the study design has been included in Fig. 5 as suggested.

• Figure 1: Consider including all between-group significances in a small table included within the image, rather than in the figure legend

To avoid presenting a too crowded figure, we prefer having the significances in the figure legend.

Minor:

• If possible, consider increasing the DPI of your tiff figures to ensure that all legends are easily readable in their final form, because owing to the small fonts, it can be hard for a curious reader to discern them, especially in the components of figure 2 (A-E)

The resolution of the legends in Fig. 2 have been increased for better readability. Furthermore, the font size has been increased for the taxonomy names labelling.

• Optionally, consider including supplementary figures alongside their respective legends in a separate PDF file to facilitate readability and continuity rather than including captions separately and uploading a zip file with images only;

Thank you for your suggestion, this has been amended accordingly.

• Consider carefully screening the text for minor English language mistakes, especially adverb utilization, tense discrepancies etc.

Minor language mistakes, mainly tense discrepancies, have been corrected throughout the manuscript.

Additional changes:

- The fecal samples were collected after 8-weeks but it was a 9-weeks intervention study. In the manuscript, the study was described as a 8-weeks intervention, which has been corrected to 9-weeks.
- Description of the harvesting of blood in the ‘Sample Collection and Processing’ section has been deleted as the blood analysis was not incl. in the manuscript.
